# Efficient Beampattern Synthesis for Sparse Frequency Diverse Array via Matrix Pencil Method

**DOI:** 10.3390/s22031042

**Published:** 2022-01-28

**Authors:** Xiaolang Shao, Taiyang Hu, Jinyu Zhang, Lei Li, Mengxuan Xiao, Zelong Xiao

**Affiliations:** School of Electronic and Optical Engineering, Nanjing University of Science and Technology, Nanjing 210094, China; xiaolangshao@njust.edu.cn (X.S.); zhangjinyu@njust.edu.cn (J.Z.); lilei220104011104@njust.edu.cn (L.L.); mengxuanxiao@njust.edu.cn (M.X.); zelongxiao@njust.edu.cn (Z.X.)

**Keywords:** frequency diverse array, array pattern synthesis, sparse array, matrix pencil method

## Abstract

Due to the introduction of frequency offsets, the pattern synthesis problem of sparse Frequency diverse array (FDA) becomes more complicated than that of the phased array. A typical way to solve this problem is to use a global optimization algorithm, but this is usually time-consuming. In this paper, we propose an efficient non-iterative beampattern synthesis approach for sparse FDA. For a given reference pattern, which can be generated by other synthesis methods, we first sample it uniformly and construct the Hankel matrix with the sampled data. By low-rank processing, a low-rank approximation version of the Hankel matrix can then be obtained. Finally, the matrix enhancement and matrix pencil (MEMP) and matrix pencil (MP) methods are applied to estimate the antenna positions, frequency offsets, and excitations of the obtained array from the approximated matrix. Besides this, two typical FDA frameworks including multi-carrier FDA (MCFDA) and standard FDA (SFDA) are considered. Numerical simulation results prove that the proposed method outperforms the existing methods in terms of synthesis error, average runtime, and percentage of saving elements.

## 1. Introduction

Frequency diverse array (FDA), as a new type of flexible scanning array, was proposed by Antonik in 2006 [1,2]. By introducing a tiny frequency offset between adjacent antenna elements, the FDA generates a range-angle-dependent steering vector. Benefiting from this feature, the FDA can provide directional gain or attenuation in range-angle space, thereby delivering potential applications in target range-angle estimation and mainlobe interference suppression [3,4,5]. Compared with phased array (PA), the FDA offers an additional design variable, i.e., frequency offset; thus, designers can obtain the desired beampattern by optimizing the frequency offset. In the past decade, numerous investigations have been developed to design frequency offset for yielding a required beampattern [6,7,8,9,10,11]. Additionally, Gao et al. proposed a multi-carrier FDA scheme, which can bring more freedom for performance improvement [12].

As a matter of fact, most of the methods mentioned above are designed in a uniform FDA framework. Despite the success of these techniques, the synthesis of uniformly spaced FDA sometimes requires a large number of elements to produce the desired pattern characteristics. However, in some cases where the weight and size of antenna systems are limited, it is very significant to obtain the desired beampattern performance with the sparse array. In the past several decades, the pattern synthesis problem of the sparse array has been extensively studied [13,14,15,16,17,18,19,20,21,22]. The typical synthesis methods of sparse arrays can be summarized into three categories: (1) global optimization algorithms [13,14,15,16]; (2) non-iterative algorithms [17,18,19]; and (3) sparsity constraint-based algorithms [20,21,22]. Nevertheless, most of these methods are designed for PAs, and the research on beampattern synthesis for sparse FDA is still lacking. Unlike the pattern synthesis for PAs, the FDA pattern synthesis should consider a new design variable, namely, frequency offset, which makes the sparse FDA synthesis more complicated.

To address this issue, Yang et al. proposed a sparse FDA design method, using the artificial bee colony (ABC) algorithm to jointly optimize the number, position, and frequency offset of antenna elements [23]. Using the convex optimization technique, a group sparse multicarrier FDA thinning method was presented in [24]. This method reduced the number of elements by turning off the antennas with very small and irrelevant weights. Chen et al. reformulated the sparse FDA synthesis problem as finding a joint sparse weight vector and placed the antenna unit corresponding to the non-zero mapping position of the joint sparse weight vector [25]. Although these works can obtain the desired pattern, they either need to be searched in high-dimensional parameter space or a time-consuming iterative process, which will bring a considerable computational burden, especially for large arrays. As far as we know, no non-iterative sparse FDA synthesis algorithm has been proposed so far.

Inspired by this observation, this paper proposes an efficient non-iterative sparse FDA beampattern synthesis method. The two typical FDA structures are considered respectively, namely, standard FDA (SFDA) and multi-carrier FDA (MCFDA). For the SFDA, we sample the desired pattern uniformly, build the block Hankel matrix with the sampled data, and perform low-rank approximate on the block Hankel matrix. The matrix enhancement matrix pencil (MEMP) method can then be applied to estimate array positions, frequency offsets, and excitations from the approximated matrix. For MCFDA, the array factor can be expressed as the product of the main cut plane of the pattern in the range and angle dimensions, and therefore we only need to consider two simple one-dimensional parameter estimation problems, which can be easily solved by matrix pencil (MP) method. Compared with the reported sparse FDA beampattern synthesis methods presented in [23,24,25], the advantages of the proposed algorithm can be summarized as (i) it is a non-iterative method and therefore computationally efficient, and (ii) the proposed method is more flexible in assigning element positions and frequency offsets, which are not restricted by the regular lattice. Numerical examples show that the proposed algorithm can accurately approximate the desired beampattern with a sparse FDA in a short time.

The structure of this paper is organized as follows. Section 2 gives the array factor model of SFDA and MCFDA. In Section 3, the proposed pattern synthesis methods for sparse SFDA and MCFDA are derived respectively. Numerical examples are performed to prove the effectiveness of the proposed algorithm in Section 4. Section 5 concludes this paper.

## 2. Array Factor of FDA

In this section, two typical FDA structures will be considered, namely, SFDA and MCFDA. Subsequently, the two FDA array factors were fully investigated.

### 2.1. Standard FDA

**Figure 1:**
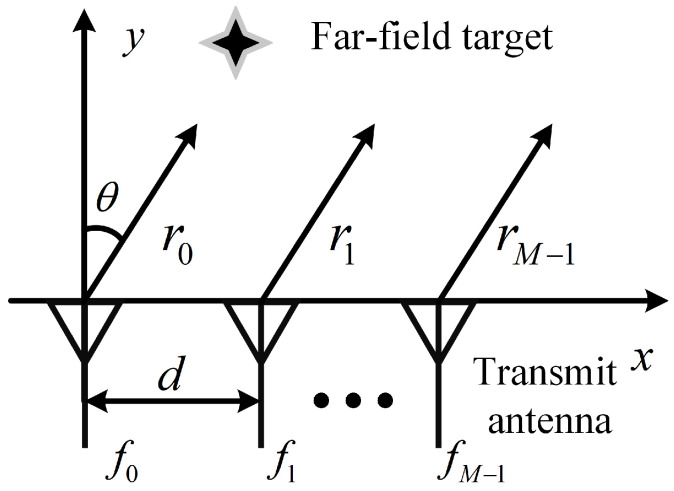
The schematic diagram of standard frequency diverse array.

In the standard FDA, a set of frequency offsets is introduced across the array, resulting in a range-angle dependent steering vector. Consider a uniform SFDA consisting of *M* antennas with adjacent spacing *d*, as shown in Figure 1. Let the first element be the reference array element, and the transmitted frequency of the *m*th antenna element can be expressed as

(1) $$f_{m}=f_{0}+\Delta f_{m},\;m=0,1,\cdots ,M-1.$$

where $f_{0}$ is the carrier frequency of the FDA system, and $\Delta f_{m}$ is the frequency offset of *m*th element. Then, the radiated signal by the *m*th antenna can be written as

(2) $$s_{m}\left(t\right)=e^{j2\pi f_{m}t}.$$

Suppose there is a target located at (θ,r) in the observation region, under far-field conditions, and the transmitted signal collected at the target can be modeled as

(3) $$\begin{array}{c} s\left(t,r,\theta \right)=\sum_{m=0}^{M-1}\omega_{m}s_{m}\left(t-\frac{r_{m}}{c}\right), \end{array}$$

where $r_{m}$ is the slope range between the target and the *m*th element, and it can be approximated as $r_{m}=r-md\sin\theta$ under far-field condition. $\omega_{m}$ and *c* represent the complex transmit weight of the *m*th element and the speed of light. Substituting Equation (2) into Equation (3), the array factor of SFDA can be formulated as

(4) $$\begin{array}{c} AF_{SFDA}\left(t,r,\theta \right)=e^{-2\pi f_{0}r/c}\sum_{m=0}^{M-1}\omega_{m}e^{j2\pi (f_{m}t+\frac{mf_{0}d\sin\theta }{c}-\frac{\Delta f_{m}r}{c}+\frac{m\Delta f_{m}d\sin\theta }{c})}. \end{array}$$

In general, the condition $\max\left|\Delta f_{m}\right|\ll f_{0}$ is satisfied in the FDA radar system; thus, the term $\exp\left\{j2\pi \frac{m\Delta f_{m}d\sin\theta }{c}\right\}$ can be ignored. Noteworthy, the time effect can be eliminated by using multi-channel mixing receiver [10], and, hence, the time-dependent term $\exp\left\{j2\pi f_{m}t\right\}$ in Equation (4) can also be eliminated. Ignore the constant term $\exp\{-2\pi f_{0}r/c\}$, and then Equation (4) can be rewritten as

(5) $$AF_{SFDA}\left(r,\theta \right)=\sum_{m=0}^{M-1}\omega_{m}e^{j2\pi (\frac{mf_{0}d\sin\theta }{c}-\frac{\Delta f_{m}r}{c})}.$$

### 2.2. Multi-Carrier FDA

**Figure 2:**
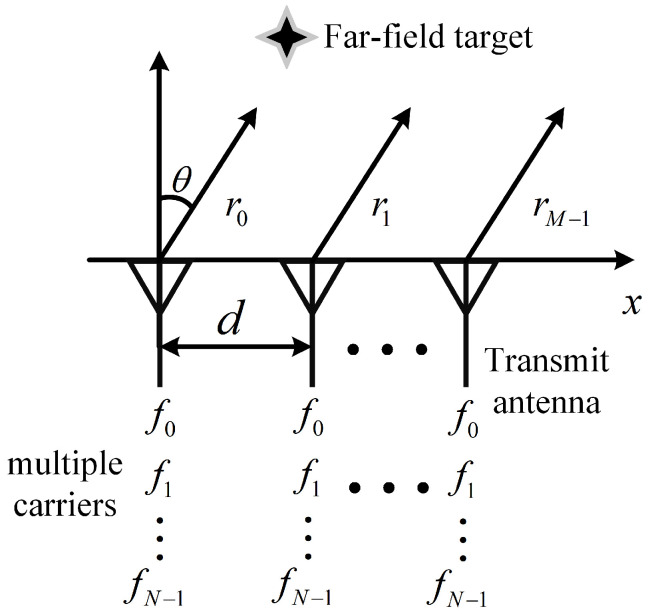
The schematic diagram of multi-carrier frequency diverse array.

In MCFDA, each array element radiates multiple carrier signals [12], thus increasing the freedom of the array. The MCFDA composed of a *M* antenna with adjacent spacing *d* is considered, as shown in Figure 2. Multiple carriers are composed of *N* frequency components, and the transmitted frequency of the *n*th carrier can be expressed as

(6) $$f_{n}=f_{0}+\Delta f_{n},\;n=0,1,\cdots ,N-1.$$

For a target (θ,r) in the far-field, the corresponding array factor for MCFDA can be expressed as

(7) $$\begin{array}{c} AF_{MCFDA}\left(t,r,\theta \right)=\sum_{m=0}^{M-1}\sum_{n=0}^{N-1}\omega_{m,n}e^{j2\pi f_{n}\left(t-r_{m}/c\right)}, \end{array}$$

where $\omega_{m,n}$ is the transmit weight of the *n*th carrier of the *m*th element. Substitute Equation (6) and $r_{m}=r-md\sin\theta$ into Equation (7) to yield

(8) $$AF_{MCFDA}\left(t,r,\theta \right)=e^{-2\pi f_{0}r/c}\sum_{m=0}^{M-1}\sum_{n=0}^{N-1}\omega_{m,n}e^{j2\pi (f_{n}t-\frac{\Delta f_{n}r}{c})}e^{j2\pi f_{0}md\sin\theta /c}.$$

Considering the common factor $\exp\{-2\pi f_{0}r/c\}$ can be ignored, and the time-dependent term $\exp\{j2\pi f_{n}t\}$ also can be canceled with the multi-channel mixing receiver. Accordingly, the final array factor of MCFDA can be expressed as

(9) $$AF_{MCFDA}\left(r,\theta \right)=\sum_{m=0}^{M-1}\sum_{n=0}^{N-1}\omega_{m,n}e^{-j2\pi \frac{\Delta f_{n}r}{c}}e^{j2\pi f_{0}md\sin\theta /c}.$$

Define $\omega_{m,n}=\omega_{m}\times \omega_{n}$ [12], then Equation (9) can be equivalent to

(10) $$AF_{MCFDA}\left(r,\theta \right)=\sum_{m=0}^{M-1}\omega_{m}e^{j2\pi f_{0}md\sin\theta /c}\sum_{n=0}^{N-1}\omega_{n}e^{-j2\pi \frac{\Delta f_{n}r}{c}}.$$

## 3. Proposed Synthesis Method for Sparse FDA

Due to the introduction of frequency offsets, the pattern synthesis problem of the FDA becomes more complicated than traditional array pattern synthesis. In the currently reported works, global optimization or convex optimization techniques have been introduced to deal with this problem [23,24,25]. However, these methods require a time-consuming search or iterative process. To alleviate this problem, an efficient non-iterative sparse FDA beampattern synthesis method is presented in this section.

### 3.1. Proposed Synthesis Method for Sparse SFDA

Consider a sparse SFDA with $\tilde{M}$ elements, by defining u=sinθ and $v=r/f_{0}$ respectively, and according to Equation (5), the array factor of sparse SFDA can be formulated as

(11) $$AF_{SFDA}^{\tilde{M}}\left(u,v\right)=\sum_{m=0}^{\tilde{M}-1}\omega_{m}e^{jk(x_{m}u-y_{m}v)},$$

where $x_{m}$ and $y_{m}$ represent the position and frequency offset corresponding to the *m*th element respectively. k=2π/λ denotes the number of waves, and $\lambda =c/f_{0}$ is the wavelength.

For a given reference beampattern $AF_{ref}\left(u,v\right)$, which can be generated by uniform SFDA with *M* array elements, the sparse MCFDA pattern synthesis problem can be equivalent to finding the minimum value $\tilde{M}$ to approximate the reference pattern.

(12) $$\begin{array}{cc} \; & \min\;\{\tilde{M}\}\\ \; & s.t.\;\left\{min\;\int \int \;{\left|AF_{ref}\left(u,v\right)-AF_{MCFDA}^{\tilde{M}}\left(u,v\right)\right|}^{2}dudv\leq \varepsilon \right\}\\ \; & \;\;\forall u\in \left[-1,1\right],v\in \left[r_{min}/f_{0},r_{max}/f_{0}\right], \end{array}$$

where ε is the reconstruction error. Based on the fact that the pattern expression is a sum of complex exponentials, the MP method is introduced to solve this question [26].

First, we need to sample the reference pattern $AF_{ref}\left(u,v\right)$ in u−v plane uniformly. Let the number of sampling points along *u* and *v* axes be 2I+1 and 2J+1 respectively, then the sample points $u_{i}$ and $v_{j}$ can be written as

(13) $$\begin{array}{cc} \; & u_{i}=i\Delta_{u},\;i=-I,-I+1,\cdots ,I\;\\ \; & v_{j}=\left(J_{s}+j\right)*\Delta_{v},\;j=0,1,\cdots ,2J, \end{array}$$

where $\Delta_{u}$ and $\Delta_{v}$ are the sampling spacing across *u* and *v* axes respectively, and $J_{s}=\frac{r_{min}}{f_{0}\Delta_{v}}$. According to the Nyquist sampling theorem, $\Delta_{u}$ and $\Delta_{v}$ should meet the conditions that $\Delta_{u}\leq \frac{\lambda }{2x_{max}}$ and $\Delta_{v}\leq \frac{\lambda }{2y_{max}}$ with $x_{max}=\max\left\{x_{m}\right\}$ and $y_{max}=\max\left\{y_{m}\right\}$. After the sampling, we have

(14) $$f\left(i,j\right)=\sum_{m=0}^{M-1}\omega_{m}p_{m}^{i}q_{m}^{j+J_{s}}$$

where $p_{m}=e^{jkx_{m}\Delta_{u}}$ and $q_{m}=e^{-jky_{m}\Delta_{v}}$. With the sampled data, the block Hankel matrix $X_{e}$ can be obtained as follows [27]:

(15) $$X_{e}=\left[\begin{array}{cccc} X_{0} & X_{1} & \cdots & X_{2I-K+1}\\ X_{1} & X_{2} & \; & X_{2I-K+2}\\ \vdots & \vdots & \ddots & \vdots \\ X_{K-1} & X_{K} & \cdots & X_{2I} \end{array}\right]$$

(16) $$X_{i}=\left[\begin{array}{cccc} f(i-I,0) & f(i-I,1) & \cdots & f(i-I,2J-L+1)\\ f(i-I,1) & f(i-I,2) & \; & f(i-I,2J-L+2)\\ \vdots & \vdots & \ddots & \vdots \\ f(i-I,L-1) & f(i-I,L) & \cdots & f(i-I,2J) \end{array}\right]$$

in which *K* and *L* are pencil parameters, which should be chosen such that KL≥M and (2I−K+2)(2J−L+2)≥M with *M* being the element number of reference pattern [18]. Then the singular value decomposition of the matrix $X_{e}$ is performed as

(17) $$X_{e}=U\Sigma V^{H}$$

where U and V are unitary matrices. $\Sigma =diag\{\sigma_{1},\sigma_{2},\cdots ,\sigma_{M},\cdots ,\sigma_{Y}\}$ with $\sigma_{m}$ being the ordered singular values of $X_{e}$, and Y=min{KL,(2I−K+2)(2J−L+2)}. Based on the observation that, for many designed antenna arrays, the number of principal singularities is less than the number of antenna elements [17], we can discard the non-principal values to obtain a low rank approximation of $X_{e}$. It is common practice to set these small singular values to zero [17,18]. That is,

(18) $$X_{\tilde{M}}=U\Sigma_{\tilde{M}}V^{H}$$

where $\Sigma_{\tilde{M}}=diag\left\{\sigma_{1},\sigma_{2},\cdots ,\sigma_{\tilde{M}},0,\cdots ,0\right\}$. The minimum value of $\tilde{M}$ can be roughly estimated as

(19) $$\tilde{M}=min\{\tilde{m};|(\sqrt{\sum_{i=\tilde{m}+1}^{M}\sigma_{i}^{2}}/\sqrt{\sum_{i=1}^{\tilde{m}}\sigma_{i}^{2}})<\varepsilon |\}.$$

Once the low-rank matrix $X_{\tilde{M}}$ is acquired, the parameters $p_{m}^{\prime }$ corresponding to the positions of the new sparse array with $\tilde{M}$ elements can be calculated by solving the following generalized eigenvalue problem [28],

(20) $$\left(X_{\tilde{M},f}-p^{\prime }X_{\tilde{M},l}\right)v=0,$$

where $X_{\tilde{M},f}$ and $X_{\tilde{M},l}$ are obtained by deleting the first *L* rows and the last *L* rows of $X_{\tilde{M}}$. Besides this, we can obtain eigenvalues more computationally efficient by solving the following equation

(21) $${\left(U_{\tilde{M},b}^{H}U_{\tilde{M},b}\right)}^{-1}U_{\tilde{M},b}^{H}U_{\tilde{M},t}-pI=0,$$

where $U_{\tilde{M},t}$ and $U_{\tilde{M},b}$ are obtained by removing the first *L* rows and the last *L* rows of $U_{\tilde{M}}$ which contain only principal right singular vectors of U. Similarly, another set of eigenvalues $q_{m}^{\prime }$ corresponding to the frequency offsets also can be obtained. Next, utilize the pairing algorithm in [28] to get the correct pairing of $p_{m}^{\prime }$ and $q_{m}^{\prime }$. Finally the locations and frequency offsets of the resulting sparse SFDA can be given by

(22) $$\begin{array}{cc} \; & x_{m}^{\prime }=\frac{\ln{\hat{p}}_{m}}{jk\Delta_{u}}.\\ \; & y_{m}^{\prime }=\frac{-\ln{\hat{q}}_{m}}{jk\Delta_{v}}. \end{array}$$

where

(23) $${\hat{p}}_{m}=\frac{p_{m}^{\prime }}{\left|p_{m}^{\prime }\right|},\;{\hat{q}}_{m}=\frac{q_{m}^{\prime }}{\left|q_{m}^{\prime }\right|}.$$

Once all the frequency offsets and element positions are available, the elements’ excitations can be calculated using the following equation

(24) $$W^{\prime }={\overset{˘}{E}}_{L}X_{e}{\overset{˘}{E}}_{R},$$

and the diagonal elements of matrix $W^{\prime }$ are $\omega_{m}^{\prime }\left(m=1,\cdots ,\tilde{M}\right)$. ${\overset{˘}{E}}_{L}$ and ${\overset{˘}{E}}_{R}$ are shown as follows:

(25) $$\begin{array}{cc} \; & {\overset{˘}{E}}_{L}={\left(E_{L}^{H}E_{L}\right)}^{-1}E_{L}^{H}\\ \; & {\overset{˘}{E}}_{R}=E_{R}^{H}{\left(E_{R}E_{R}^{H}\right)}^{-1}, \end{array}$$

where

(26) $$E_{L}=\left[\begin{array}{c} Q_{L}P_{d}^{-I}\\ Q_{L}P_{d}^{-I+1}\\ \vdots \\ Q_{L}P_{d}^{K-I-1} \end{array}\right]$$

(27) $$E_{R}=\left[\begin{array}{cccc} Q_{R}, & P_{d}Q_{R}, & \cdots , & P_{d}^{2I-K-1}Q_{R} \end{array}\right]$$

wherein

(28) $$Q_{L}=\left[\begin{array}{cccc} {\hat{q}}_{1}^{J_{s}} & {\hat{q}}_{2}^{J_{s}} & \cdots & {\hat{q}}_{\tilde{M}}^{J_{s}}\\ {\hat{q}}_{1}^{J_{s}+1} & {\hat{q}}_{2}^{J_{s}+1} & \cdots & {\hat{q}}_{\tilde{M}}^{J_{s}+1}\\ \vdots & \vdots & \vdots & \vdots \\ {\hat{q}}_{1}^{J_{s}+L-1} & {\hat{q}}_{2}^{J_{s}+L-1} & \cdots & {\hat{q}}_{\tilde{M}}^{J_{s}+L-1} \end{array}\right]$$

(29) $$P_{d}=diag\left\{{\hat{p}}_{1},{\hat{p}}_{2},\cdots ,{\hat{p}}_{\tilde{M}}\right\}$$

(30) $$Q_{R}=\left[\begin{array}{cccc} 1 & {\hat{q}}_{1} & \cdots & {\hat{q}}_{1}^{2J-L+1}\\ 1 & {\hat{q}}_{2} & \cdots & {\hat{q}}_{2}^{2J-L+1}\\ \vdots & \vdots & \vdots & \vdots \\ 1 & {\hat{q}}_{\tilde{M}} & \cdots & {\hat{q}}_{\tilde{M}}^{2J-L+1} \end{array}\right]$$

The steps are summarized in Algorithm 1.
**Algorithm 1:** Proposed synthesis method for sparse FDA.**Input:** $AF_{ref}\left(u,v\right),I,J,K,L,\varepsilon$1: Sample reference pattern in u−v plane uniformly according to Equations (13) and (14), and  construct the block Hankel matrix $X_{e}$ using Equations (15) and (16).2: Perform the singular value decomposition (SVD) of $X_{e}$ according to Equation (17) and calculate the singular values Σ.3: According to Equation (19), determine the minimum number of elements $\tilde{M}$.4: According to Equation (21), extract the eigenvalues ${p^{\prime }}_{m}$ and ${q^{\prime }}_{m}$, then to pair them utilizing pairing algorithm in [28].5: Detemine frequency offsets $x_{m}^{\prime }$ and locations $y_{m}^{\prime }$ of the new sparse array with $\tilde{M}$ using Equations (22) and (23)6: Calculate the excitations $\omega_{m}^{\prime }$ using Equations (24)–(30).**Output:** $\omega_{m}^{\prime },x_{m}^{\prime },y_{m}^{\prime }$

In Algorithm 1, the most computationally intensive operations mainly include the SVD of the block Hankel matrix with $O(KL{\left(2I-K+2\right)}^{2}{\left(2J-L+2\right)}^{2})$ in step 2 and the eigenvalue decomposition (ED) with $O({\tilde{M}}^{3})$ in step 4. Therefore, the total computational complexity is $O(KL{\left(2I-K+2\right)}^{2}{\left(2J-L+2\right)}^{2}+{\tilde{M}}^{3})$. For the pattern synthesis problem of sparse SFDA, the typical method is to adopt global optimization algorithms, e.g., [23]. Since the number of iterations guaranteed to converge is hard to know, it is difficult to compare in terms of computational complexity. However, according to the authors’ observations, the average execution time of the proposed method is much lower than that of [23].

### 3.2. Proposed Synthesis Method for Sparse MCFDA

Suppose a sparse MCFDA consists of $\tilde{M}$ antenna elements with $\tilde{N}$ carriers, and according to Equation (10), the array factor of sparse MCFDA can be expressed as

(31) $$\begin{array}{cc} AF_{MCFDA}^{\left\{\tilde{M},\tilde{N}\right\}}\left(u,v\right) & =\sum_{m=0}^{\tilde{M}-1}\omega_{m}e^{jkx_{m}u}\sum_{n=0}^{\tilde{N}-1}\omega_{n}e^{-jky_{n}v}, \end{array}$$

where $x_{m}$ and $y_{n}$ represent the position corresponding to the *m*th element and frequency offset corresponding to the *n*th carrier, respectively. According to Equation (12), the pattern synthesis problem for sparse MCFDA can also be described mathematically as

(32) $$\begin{array}{cc} \; & \min\;\{\tilde{M},\tilde{N}\}\\ \; & s.t.\;\left\{min\;\int \int \;{\left|AF_{ref}\left(u,v\right)-AF_{MCFDA}^{\left\{\tilde{M},\tilde{N}\right\}}\left(u,v\right)\right|}^{2}dudv\leq \varepsilon \right\}\\ \; & \;\;\forall u\in \left[-1,1\right],v\in \left[r_{min}/f_{0},r_{max}/f_{0}\right]. \end{array}$$

Besides this, it can be seen from Equation (31) that the array factor of MCFDA can be decomposed as the product of two individual exponential summations corresponding to *u* and *v*. Accordingly, Equation (32) can further be recast as [25]

(33) $$\begin{array}{cc} \; & \min\;\{\tilde{M},\tilde{N}\}\\ \; & s.t.\left\{min∥AF_{ref}^{\theta }\left(u\right)-AF_{MCFDA}^{\left\{\tilde{M}\right\}}\left(u\right)∥\right\}\leq \varepsilon_{u}\\ \; & \;\left\{min∥AF_{ref}^{r}\left(v\right)-AF_{MCFDA}^{\left\{\tilde{N}\right\}}\left(v\right)∥\right\}\leq \varepsilon_{v}, \end{array}$$

where $AF_{ref}^{\theta }\left(u\right)$ and $AF_{ref}^{r}\left(v\right)$ are the cross section of reference beampattern $AF_{ref}\left(u,v\right)$ along the *u* and *v* axes, respectively. $\varepsilon_{u}$ and $\varepsilon_{v}$ are the reconstructed error threshold, and $AF_{MCFDA}^{\left\{\tilde{N}\right\}}\left(v\right)$ and $AF_{MCFDA}^{\left\{\tilde{M}\right\}}\left(u\right)$ are given by

(34) $$\begin{array}{c} AF_{MCFDA}^{\left\{\tilde{M}\right\}}\left(u\right)=\sum_{m=0}^{\tilde{M}-1}\omega_{m}e^{jkx_{m}u}\\ AF_{MCFDA}^{\left\{\tilde{N}\right\}}\left(v\right)=\sum_{n=0}^{\tilde{N}-1}\omega_{n}e^{-jky_{n}v}. \end{array}$$

It is observed that the synthesis of sparse MCFDA can be translated into two independent sparse array pattern synthesis problems which depend on the array positions and frequency offsets, respectively. Therefore, we only need to consider two simple one-dimensional parameter estimation problems, which can be easily solved by MPM.

Similarly, we sample the reference pattern $AF_{ref}^{\theta }\left(u\right)$ and $AF_{ref}^{r}\left(v\right)$ respectively, constructe the Hanke matrix $X_{u}$ and $X_{v}$ by the sampling points as follows, and perform the singular value decomposition of $X_{u}$ and $X_{v}$.

(35) $$X_{u}=\left[\begin{array}{cccc} f_{u}\left(-I\right) & f_{u}\left(-I+1\right) & \cdots & f_{u}\left(K_{u}-I\right)\\ f_{u}\left(-I+1\right) & f_{u}\left(-I+2\right) & \; & f_{u}\left(K_{u}+1-I\right)\\ \vdots & \vdots & \ddots & \vdots \\ f_{u}\left(I-K_{u}\right) & f_{u}\left(I-K_{u}+1\right) & \cdots & f_{u}\left(2I\right) \end{array}\right]$$

(36) $$X_{v}=\left[\begin{array}{cccc} f_{v}\left(0\right) & f(1) & \cdots & f(K_{v})\\ f_{v}\left(1\right) & f(2) & \; & f(K_{v}+1)\\ \vdots & \vdots & \ddots & \vdots \\ f_{v}\left(2J-K_{v}\right) & f(j) & \cdots & f(2J) \end{array}\right],$$

where $f_{u}\left(u\right)$ and $f_{v}\left(v\right)$ are the sampled points obtained from $AF_{ref}^{\theta }\left(u\right)$ and $AF_{ref}^{r}\left(v\right)$, and $K_{u}$ and $K_{v}$ are the pencil parameters. Generally, $K_{u}$ and $K_{v}$ should satisfy the conditions $K_{u}\geq M$ and $K_{v}\geq N$ with *M* and *N* being the number of elements of the reference pattern [17]. Then, the minimum value $\left\{\tilde{M},\tilde{N}\right\}$ and the reconstructed low-rank matrix $X_{u}^{\tilde{M}}$ and $X_{v}^{\tilde{N}}$ can be acquired using Equations (18) and (19). The parameters $p_{m}^{\prime }$ and $q_{m}^{\prime }$ can be obtained by solving the following equations [17].

(37) $$\begin{array}{ccc} \; & {\left(V_{\tilde{M},b}^{H}V_{\tilde{M},b}\right)}^{-1}V_{\tilde{M},b}^{H}U_{\tilde{M},t}-pI=0,\; & {\left(V_{\tilde{N},b}^{H}V_{\tilde{N},b}\right)}^{-1}V_{\tilde{N},b}^{H}V_{\tilde{N},t}-qI=0, \end{array}$$

where $V_{\tilde{M},t}$ (resp.,$V_{\tilde{M},b}$) and $V_{\tilde{N},t}$ (resp.,$V_{\tilde{N},b}$) are obtained by deleting the top (resp.,) row of $V^{\tilde{M}}$ and $V^{\tilde{N}}$, which consist of $\tilde{M}$ and $\tilde{N}$ principal left-singular vectors. Next, the locations $x_{m}^{\prime }$ and frequency offsets $y_{m}^{\prime }$ of the new sparse MCFDA can be obtained by Equation (22). It should be noted that the pairing operation is not required. Finally, the excitations $\omega_{m}$ and $\omega_{n}$ can be calculated by the least squares (LS) method. The implementation steps of sparse MCFDA are listed in Algorithm 2.
**Algorithm 2:** Proposed synthesis method for sparse MCFDA.**Input:** $AF_{ref}^{\theta }\left(u\right),AF_{ref}^{\theta }\left(v\right),I,J,K,L,\varepsilon_{u},\varepsilon_{v}$1: Sample two desired patterns $AF_{ref}^{\theta }\left(u\right)$ and $AF_{ref}^{\theta }\left(v\right)$ respectively, and construct the Hankel matrix $X_{u}$ and $X_{v}$ using Equations (35) and (36).2: Perform the SVD of $X_{u}$ and $X_{v}$ and determine the minimum number value $\tilde{M}$ and $\tilde{N}$.3: According to Equation (37), extract the eigenvalues ${p^{\prime }}_{m}$ and ${q^{\prime }}_{n}$.4: Detemine locations $x_{m}^{\prime }$ and carriers $y_{n}^{\prime }$ of the new sparse MCFDA using Equations (22) and (23)5: Calculate the excitations $\omega_{m}^{\prime }$ and $\omega_{n}^{\prime }$ using the LS method.**Output:** $\omega_{m}^{\prime },x_{m}^{\prime },\omega_{n}^{\prime },y_{m}^{\prime }$

Similar to the analysis of Algorithm 1, the computational complexity of Algorithm 2 is close to $O(\left(2I-Ku+1\right){\left(Ku+1\right)}^{2}+\left(2J-Kv+1\right){\left(Kv+1\right)}^{2}+{\tilde{M}}^{3}+{\tilde{N}}^{3})$. For the synthesis problem of sparse MCFDA, we adopt the method presented in [25] as comparison. The computational complexity of the method in [25] is $O(N_{i}^{3.5})$, where $N_{i}$ is the number of the initial dense array. In general, the conditions $N_{i}\geq \max\left\{2I-Ku+1,Ku+1\right\}$ and $N_{i}\geq \max\left\{2J-Kv+1,Kv+1\right\}$ are satisfied. Consequently, the complexity of the proposed algorithm is lower than that of [25].

## 4. Results and Discussions

In order to verify the effectiveness of the proposed algorithm, two numerical examples are given in this section. The reference patterns are generated utilizing the previously reported FDA pattern synthesis methods [3,10]. The normalized matching error ξ is used for evaluation, as shown in following

(38) $$\xi =\frac{\;\int \int \;{\left|AF_{ref}\left(u,v\right)-AF_{rec}\left(u,v\right)\right|}^{2}dudv}{\;\int \int \;{\left|AF_{ref}\left(u,v\right)\right|}^{2}dudv},$$

where $AF_{rec}\left(u,v\right)$ is the reconstructed pattern.

### 4.1. Example 1: Beampattern Synthesis for Sparse SFDA

In this example, the SFDA scheme with symmetric logarithmically increasing frequency offsets presented in [10] is adopted to generate the reference pattern. The parameters of the uniform SFDA are set as $f_{0}=10$ GHz, $d=c/f_{0}=0.015$ m, N=20, and Δf=20 kHz. Moreover, The observation region is defined as Ω:{30 km ≤r≤70 km, $-90^{\circ }\leq \theta \leq 90^{\circ }\}$ and the single target is located at (50 km, $0^{\circ })$. Notice that the parameters are set to I=J=2M, which is sufficient to accurately approximate the reference beampattern, and the pencil parameters are set as K=L=2M+1. Besides this, several published works have shown that $\varepsilon =10^{-2}$ allowed excellent reconstructions [17,29]. Furthermore, to verify the superiority of the proposed algorithm, we adopt the synthesis method presented in [23] as a comparison in the same simulation conditions. It should be mentioned that we need to modify the cost function in [23] to suit our task. More specifically, the cost function should be replaced by Equation (12).

**Figure 3:**
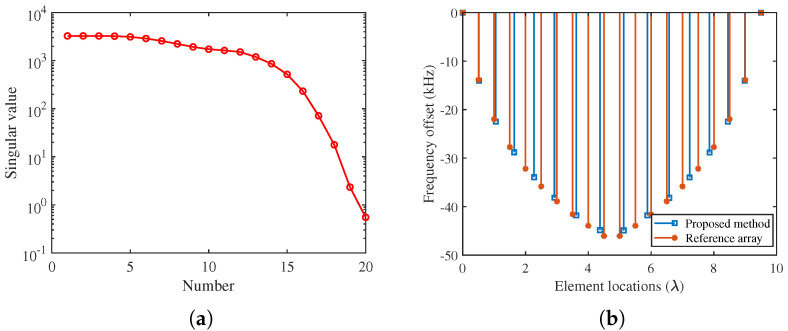
The eigenvalue spectrum and the distribution of frequency offsets and element positions. (**a**) The eigenvalue spectrum of the reference pattern. (**b**) The distribution of frequency offsets and element positions.

Figure 3a shows the eigenvalue spectrum of the reference pattern. It is noted that the singular values exceeding the 14th value decay rapidly, which means the very small eigenvalues can be ignored. Consequently, the reference pattern can be reconstructed with fewer array elements. In our case, the minimum estimated value $\tilde{M}$ is 16 at $\varepsilon =10^{-2}$. The comparison of the element positions and frequency offsets between the original array and the obtained sparse array is given in Figure 3b. It can be observed that the minimum spacing of adjacent elements in the obtained array is greater than λ/2 without specific constraints, which is conducive to reducing the mutual coupling effect. Notably, unlike other methods presented in [23], the proposed method does not force the antennas to be deployed as an integer multiple of a fixed spacing, allowing it has the potential of approximating the reference pattern with fewer elements. Besides this, the new sparse SFDA has a slightly smaller total frequency offset than the uniform SFDA.

**Figure 4:**
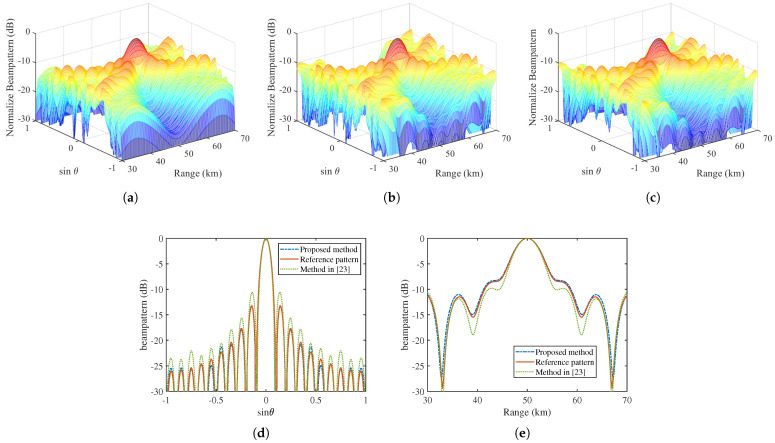
The comparison of patterns generated by different sparse SFDA methods. (**a**) The 3D reference pattern. (**b**) The 3D reconstructed pattern obtained by using the method in [23]. (**c**) The 3D reconstructed pattern obtained by using proposed method. (**d**) The cross section of the pattern along angle dimension. (**e**) The cross section of the pattern along range dimension.

The comparison of patterns generated by different sparse SFDA methods is depicted in Figure 4. Obviously, the sparse SFDA with 16 antennas obtained by the proposed method can well approximate the cross section of reference pattern with 20 antennas, both along the angle and range dimensions. Therefore, the array elements are saved by about 20% ($\frac{20-16}{20}\times 100\%=20\%$) by the proposed method. Table 1 shows the performance comparison of different sparse SFDA synthesis methods. It can be seen that, using our method, the normalized matching error ξ calculated by (38) is $9.5\times 10^{-3}$, which is better than the method presented in [23]. Moreover, the proposed method requires only about 2 s to complete the whole synthesis process in the computer with Intel Core i7-10875H CPU @ 2.30 GHz and 16GB RAM, while the algorithm in [23] requires about 440 s. Thus, the proposed method outperforms the method in [23] in terms of pattern matching accuracy and time consumption. In addition, the array positions, excitations, and frequency offsets of the obtained sparse SFDA are listed in Table 2. Generally, the dynamic range ratio of excitations, defined as the ratio between the maximum and minimum excitation amplitude, is used to evaluate the difficulty of the excitation implementation. In our work, without specific constraints, the dynamic range ratio of the excitation is 1.5418, which can be easily implemented with the available hardware conditions.

### 4.2. Example 2: Beampattern Synthesis for Sparse MCFDA

In the second example, we consider the beampattern synthesis for a sparse MCFDA. The reference pattern is obtained by the two-dimensional discrete spheroidal sequence (DSS) method presented in [3]. The parameters of reference array are set as M=N=32, Δf=30 kHz, f0=10 GHz and d=0.015 m. The visible region is set as Ω: {6 km ≤r≤14 km, $-90^{\circ }\leq \theta \leq 90^{\circ }\}$ and the single target is steered to (10 km, $0^{\circ })$. Moreover, the parameters $I=K_{u}=2M$, $J=K_{v}=2N$ and $\varepsilon_{u}=\varepsilon_{v}=10^{-2}.$ Additionally, the reconstructed pattern provided by the method in [25] is given for comparison.

**Figure 5:**
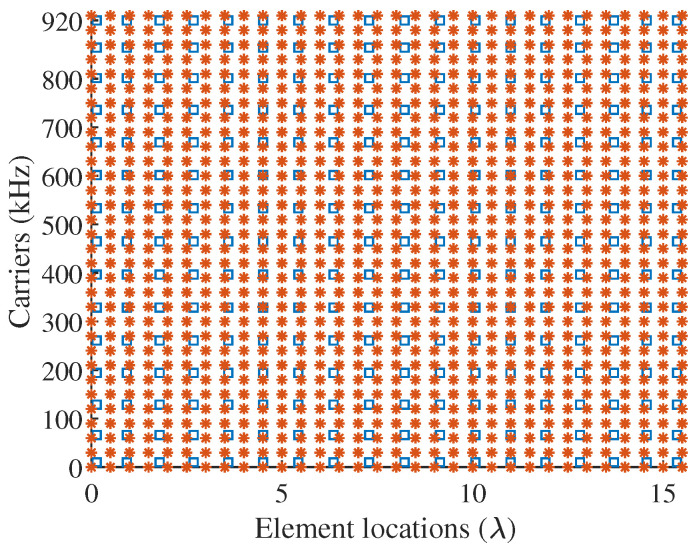
The comparsion of layout between the uniform MCFDA (red asterisk) and obtained sparse MCFDA (blue square box).

**Figure 6:**
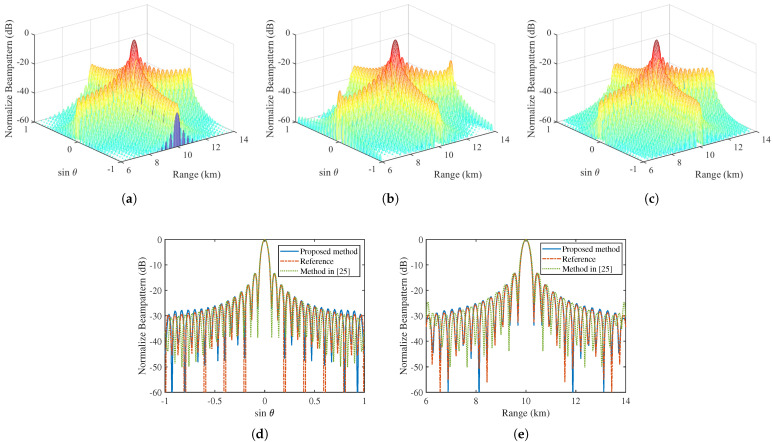
The comparison between the reconstructed pattern and reference pattern. (**a**) The 3D reference pattern. (**b**) The 3D reconstructed pattern obtained by using the method in [25]. (**c**) The 3D reconstructed pattern obtained by using proposed method. (**d**) The cross section of the pattern along angle dimension. (**e**) The cross section of the pattern along range dimension.

According to (19), the estimated minimum number of elements and carriers are $\tilde{M}=18$ and $\tilde{N}=15$, respectively. This implies that we can use a sparse MCFDA with 18 array elements and 15 carriers to reconstructe the reference pattern in a given tolerance. Figure 5 gives the comparison of layout between the uniform MCFDA and obtained sparse MCFDA. It is observed that the new sparse MCFDA obtained by our method saves about 44% ($\frac{32-18}{32}\times 100\%\approx 44\%$) of antennas and about 53% ($\frac{32-15}{32}\times 100\%\approx 53\%$) of carriers with respect to the uniform MCFDA, which is beneficial to reduce the complexity, cost, and weight of the antenna system. Conversely, the sparse MCFDA produced by the method in [25] consists of 29 elements and 29 carriers, which saves only 9.38% of the number of elements and carriers. The comparison of patterns provided by different methods is presented in Figure 6. It can be seen that the proposed method can accurately match the reference pattern in most observation areas, with only a slight deterioration of the sidelobe level. It should be noted that the matching accuracy in the sidelobes can be improved by increasing the number of antennas $\tilde{M}$ and the number of carriers $\tilde{N}$.

Table 3 shows the performance comparison of different sparse MCFDA synthesis methods. As can be seen, the normalized matching error ξ of the reconstructed pattern generated by our method is $7.7\times 10^{-4}$, which is much better than the $8.2\times 10^{-2}$ of method in [25]. It is worth mentioning that the single runtime of our method is only 0.6 s, which is also better than the 4.8 s of method in [25]. Moreover, the positions, excitations, and frequency offsets calculated by the proposed method are shown in Table 4. Note that no phase information of $\omega_{m}^{\prime }$ is provided in Table 4 since the weights $\omega_{m}^{\prime }$ are real. The dynamic range ratio of the excitations in the resultant array is 1.7464, which also can be easily implemented in practice. Similarly, the obtained sparse MCFDA yields a smaller aperture and total frequency offset.

## 5. Conclusions

In this paper, an efficient beampattern synthesis method for sparse FDA has been presented. By performing the low-rank approximation for the Hankel matrix constructed by the desired pattern samples, we can determine the minimum number of antennas for generating a reference pattern within tolerance. Then, using the MP or MEMP methods, the element positions, excitations, and frequency offsets can be obtained analytically from the low-rank version of the Hanke matrix. Besides this, two typical FDA schemes including SFDA and MCFDA are considered. The numerical examples have demonstrated that the proposed method can well approximate the desired pattern with a reduced array. In the provided examples, the entire synthesis process of the proposed algorithm only takes about a few seconds, which is very difficult for other sparse FDA synthesis methods, such as global optimization algorithms. This work concentrates more on linear FDA, and future work will focus on the development of efficient pattern synthesis methods for sparse planar FDA and circular FDA.

## Figures and Tables

**Table 1:** The performance comparison of different sparse SFDA synthesis methods.

Method	Normalized Matching Error	Percentage of Saving Elements	Average Runtime
Proposed method	$9.5\times 10^{-3}$	20%	2 s
Method in [23]	$2.59\times 10^{-2}$	20%	440 s

**Table 2:** The array positions, excitations, and frequency offsets of the obtained sparse SFDA.

Index	Locations (λ)	Frequecy Offset (kHz)	Amp. of Weights	Phase of Weights (deg)
1	0	−0.0101	0.6486	−0.6086
2	0.5160	−14.0468	0.6692	−122.8095
3	1.0539	−22.4825	0.7164	91.0485
4	1.6387	−28.8050	0.7834	71.7027
5	2.2710	−33.9395	0.8431	123.6288
6	2.9208	−38.1580	0.8789	−129.4829
7	3.6178	−41.7897	0.9184	12.6200
8	4.3720	−44.8412	1	−170.4712
9	5.1280	−44.9088	0.9965	−174.5252
10	5.8822	−41.8300	0.9190	10.2003
11	6.5792	−38.1813	0.8796	−130.8790
12	7.2290	−33.9610	0.8435	122.3387
13	7.8616	−28.8221	0.7837	70.6733
14	8.4462	−22.4928	0.7176	90.4345
15	8.9841	−14.0525	0.6694	−123.1471
16	9.4994	−0.0104	0.6486	−0.6244

**Table 3:** The performance comparison of different sparse MCFDA synthesis methods.

Method	Normalized Matching Error	Percentage of Saving Elements	Average Runtime
Proposed method	$7.7\times 10^{-4}$	44% (antenna elements), 53% (carriers)	0.6 s
Method in [25]	$8.2\times 10^{-2}$	9.38% (antenna elements), 9.38% (carriers)	4.8 s

**Table 4:** The positions, excitations, and frequency offsets of the obtained sparse MCFDA.

Index	Locations (λ)	Amp. of Weights $\omega_{m}^{\prime }$	Frequency Offsets (kHz)	Amp. of Weights $\omega_{n}^{\prime }$	Phase of Weights $\omega_{n}^{\prime }$ (deg)
1	0.1364	0.7847	10.0807	0.7297	119.9415
2	0.9292	0.9001	66.1003	0.8837	72.2399
3	1.7896	0.9476	128.7028	0.9423	103.5396
4	2.6805	0.9698	194.1029	0.9702	168.4138
5	3.5878	0.9829	260.9439	0.9855	−109.4196
6	4.5050	0.9908	328.5951	0.9941	−17.5296
7	5.4283	0.9958	396.6961	0.9986	79.7576
8	6.3553	0.9987	465.0000	1.0000	179.4811
9	7.2844	1.0000	533.3039	0.9986	−80.7955
10	8.2141	1.0000	601.4049	0.9941	16.4917
11	9.1432	0.9987	669.0561	0.9855	108.3817
12	10.0703	0.9958	735.8971	0.9702	−169.4516
13	10.9936	0.9908	801.2972	0.9423	−104.5774
14	11.9107	0.9829	863.8997	0.8837	−73.2778
15	12.8181	0.9698	919.9193	0.7297	−120.9794
16	13.7090	0.9467			
17	14.5694	0.9001			
18	15.3622	0.7847			

## Data Availability

Not applicable.
